# Evaluation of the association between the common E469K polymorphism in the ICAM-1 gene and diabetic nephropathy among type 1 diabetic patients in GoKinD population

**DOI:** 10.1186/1471-2350-9-47

**Published:** 2008-05-27

**Authors:** Jun Ma, Dongying Zhang, Kerstin Brismar, Suad Efendic, Harvest F Gu

**Affiliations:** 1 Rolf Luft Center for Diabetes Research, Department of Molecular Medicine and Surgery, Karolinska Institutet, Karolinska University Hospital, Stockholm, Sweden

## Abstract

**Background:**

The ICAM-1 gene is a strong positional and biological candidate for susceptibility to the development of T1D and DN. We have recently demonstrated that SNP rs5498(E469K) confers susceptibility to the development of T1D and might be associated with DN in Swedish Caucasians. The present study aimed to further evaluate the association between the ICAM-1 genetic polymorphisms and DN.

**Methods:**

Two common non-synonymous SNPs, including rs5498(E469K) and rs1799969(R241G), in the ICAM-1 gene were genotyped in 662 (312 female/350 male) T1D patients with DN and 620 (369/251) without DN. All patients were selected from the GoKinD study.

**Results:**

Genotype distributions of both SNPs were in Hardy-Weinberg equilibrium but SNP rs5498(E469K) had high heterozygous index. In this SNP, the heterozygosity and positivity for the allele G were found to be significantly associated with DN in female T1D patients (P = 0.010, OR = 0.633, CI 95% 0.447–0.895 and P = 0.026, OR = 0.692, CI 95% 0.500–0.958). Furthermore, the female patients without DN carrying three genotypes A/A, A/G and G/G had different cystatin levels (0.79 ± 0.17, 0.81 ± 0.14 and 0.75 ± 0.12 mg/L, P = 0.021). No significant association of SNP rs1799969 (R241G) with DN was found.

**Conclusion:**

The present study provides further evidence that SNP rs5498(E469K) in the ICAM-1 gene presents a high heterozygous index and the allele G of this polymorphism may confers the decreased risk susceptibility to the development of DN in female T1D patients among the GoKinD population.

## Background

Genome-wide scans and linkage analyses have predicted that the susceptibility gene(s) for type 1 diabetes (T1D) and diabetic nephropathy (DN) reside in chromosome 19p13 [1-3]. The intercellular adhesion molecule-1 (ICAM-1) gene is located in this chromosomal region. ICAM-1 is a 90-kD cell surface glycoprotein of the Ig super family involved in the firm attachment of leukocytes to endothelium [4,5]. Expression of ICAM-1 can be induced by multiple factors, including inflammatory cytokines, reactive oxygen species and shear stress [6,7]. Normally, ICAM-1 is expressed at low levels on the surface of arterial endothelial cells, but the expression at both mRNA and protein levels are significantly increased in animal models of diabetic nephropathy with T1D [7-9] and T2D [10-12]. Furthermore, clinical evidence indicates that the levels of plasma adhesion molecule are elevated in T1D patients [13]. Therefore, the ICAM-1 gene is considered as a strong positional and biological candidate for the susceptibility to the development of T1D and DN.

In the recent years, several genetic association studies concerning two common non-synonymous SNPs, including rs5498 E469K(A/G) and rs1799969 R241G(G/A), in the ICAM-1 gene in T1D patients have been reported. The G allele of SNP rs5498 E469K(A/G) was preferentially transmitted the affected offspring among Romanian T1D families [14]. This polymorphism was subsequently found to be associated with adult-onset T1D patients in Japanese [15]. However, the association of this SNP with T1D was not detectable in Danish, Finnish and British Caucasians [16,17]. In another SNP R241G(G/A), transmission of the allele G was also preferentially transmitted to the affected offspring among T1D families in Finnish, British, Romanian, European and American Caucasians [18]. We have recently conducted a genetic association study of five SNPs in the ICAM-1 gene among Swedish non-diabetic subjects, T1D patients with and without DN [19]. Data indicate that SNP rs5498 E469K(A/G) is associated with T1D in Swedish population. The frequency of the major A allele of this SNP is increased gradually from non-diabetic controls, to T1D patients without DN and to the patients with DN, which suggests that this SNP might be associated with DN. In order to further evaluate the susceptibility of the ICAM-1 genetic polymorphisms in the development of DN, in the present study, we have focused on genetic association analyses of two common non-synonymous SNPs i.e. rs5498 E469K(A/G) and rs1799969 R241G(G/A) in the ICAM-1 gene by using the well-selected and characterized cohort of T1D patients with and without DN from the Genetics of Kidneys in Diabetes (GoKinD) study [20].

## Methods

### Clinical material

Clinical material employed in the present study includes a cohort of 1282 (female 681/male 601) T1D patients. They were selected from the GoKinD study and collected by the Juvenile Diabetes Research Foundation in collaboration with the Joslin Diabetes Center and George Washington University, and the United States Centers for Diabetes Control and Prevention [20]. The patients with T1D were diagnosed before age 31 years old and the duration of T1D is more than 10 years. Medical treatment with insulin for the patients was instituted within one year of diagnosis, and was uninterrupted since then.

In total, 620 patients (369/251) without DN were used as the controls and 662 (312/350) with DN as the cases. Among the patients, 1177 (91.8%) were of European descent, while 105 (8.2%) patients were Americans of Black, Asian, Hispanic or Indian descent. The patients with DN had T1D for at least 10 years and had persistent proteinuria or ESRD (not due to condition other than diabetes). Persistent proteinuria was defined as 2 out of 3 tests positive for albuminuria (at least 1 month apart), i.e. dipstick (Albustix or Multistix) at least 1+ or Urine Albumin/Urine Creatinine Ratio (ACR) value exceeding 300 μg albumin/mg of urine creatinine. The patients without DN had persistent normalbuminuria despite duration of T1D for at least 15 years and had never been treated with ACE inhibitors. Persistent normoalbuminuria was defined as at least 2 out 3 of ACR measurements (at least 1 month apart) in random urine specimens being less than 20 μg albumin/mg of urine creatinine. If 3 ACR measurements were needed, the highest must also be less than 40 μg albumin/mg of urine creatinine. ESRD is recognized as the patients need chronic dialysis or kidney transplant treatment to maintain their life. The onset of ESRD is defined as the date of the first dialysis or kidney transplant, whichever occurred first. Other kidney diseases in cases were excluded [20].

Individuals were excluded from the study if they did not meet the inclusion criteria just described or if any of the following exclusion criteria were met: Unable or unwilling to give informed consent; Unable to communicate with staff; Major psychiatric disorder such as schizophrenia; Exclusion in relation to medication: Any antihypertensive medication for controls; Infectious disease: self-reported HIV positive and active tuberculosis; Pregnant women (although they may be reconsidered 3 months after delivery); Other kidney disease due to condition other than diabetes.

The cases and controls selected from the GoKinD study were T1D patients with the severity of DN and T1D patients without DN. T1D patients with micro-albuminuria were not included. The GoKinD study was approved by the local ethics committees. Material and data transfer agreement (MTA) was made prior to the present study. The clinical characteristics of the patients are presented in Table 1.

**Table 1:** Clinical characteristics of T1D patients with and without DN

	T1D patients without DN			T1D patients with DN		
		
	All	Male	Female	All	Male	Female
N	620	251	369	662	350	312
Age (yrs)	40 ± 8	40 ± 8	40 ± 9	44 ± 7	44 ± 6	43 ± 7
Duration (yrs)	26 ± 8	26 ± 8	26 ± 8	31 ± 8	32 ± 8	31 ± 8
HbA1c (%)	7.48 ± 1.14	7.40 ± 1.14	7.53 ± 1.13	7.40 ± 1.91	7.44 ± 1.74	7.35 ± 2.08
BMI (kg/m^2^)	26.1 ± 4.3	26.6 ± 3.7	25.6 ± 4.6	25.8 ± 5.4	26.1 ± 4.9	25.5 ± 5.9
Cystatin (mg/L)	0.81 ± 0.14	0.84 ± 0.12	0.79 ± 0.14	2.32 ± 1.77	2.36 ± 1.70	2.27 ± 1.84
Creatinine (mg/dL)	0.90 ± 0.16	0.99 ± 0.14	0.83 ± 0.14	2.19 ± 2.01	2.31 ± 1.96	2.05 ± 2.05
Urine albumin (μg)*	6.7 ± 4.3	6.5 ± 4.3	6.9 ± 4.3	1430 ± 1280	1357 ± 1193	1529 ± 1392
Cholesterol (mg/dL)	185.0 ± 31.5	179.7 ± 30.9	188.8 ± 31.3	186.9 ± 46.1	184.5 ± 46.2	189.7 ± 45.9
HDL (mg/dL)	59.0 ± 15.8	50.9 ± 12.4	64.7 ± 15.4	53.6 ± 17.5	48.9 ± 15.2	58.9 ± 18.5
SBP (mmHg)	118 ± 12	122 ± 12	116 ± 12	132 ± 19	134 ± 18	130 ± 20
DBP (mmHg)	71 ± 8	74 ± 8	70 ± 8	74 ± 11	76 ± 11	72 ± 11

Data are means ± SD. T1D = type 1 diabetic patients and DN = diabetic nephropathy. *Data were calculated from ACR (urine albumin creatinine ration) in T1D patients with and without DN. The patients with ESRD were not included.

### SNPs selection and genotyping

In the present study, we selected two non-synonymous SNPs, rs1799969 R241G and rs5498 E469K(A/G) for genotyping [19]. These two polymorphisms are located, respectively, in exons 4 and 6 of the ICAM-1 gene. The SNP ID numbers and detailed sequence information are available in the public SNP database [21] Genomic DNA samples were supplied from the GoKinD study. Genotyping experiments were performed using a high-throughput SNP scoring technique called dynamic allele specific hybridization (DASH). PCR-DASH assays and genotyping protocols are described in our previous report [19].

### Statistical analyses

Allele frequency and genotype distribution of the studied SNPs were tested for Hardy-Weinberg equilibrium (HWE). For differences between T1D patients with DN (the cases) and without DN (the controls), two models were tested comparing either allele frequencies in 2 × 2 contingency tables or genotypes in 3 × 2 contingency tables. The models were further used for testing either heterozygosity (22 vs 12) or allele positivity (11+12 vs 22). Odds ratios (OR) and 95% confidence intervals (CI) were calculated to test for relative risk for association. *P*-values less than 0.05 were interpreted as statistically significant. Statistical powers were calculated using software of PowerSampleSize (PS version 2.1.31) [22]. Tests for association between genotypes and quantitative traits were performed using Kruskal-Wallis analysis of ranks for traits with non-normal distributions, or alternatively, ANOVA for normally distributed traits. Normal probability plots were created and parameter distributions were transformed to natural logarithm when needed to improve the skew-ness and for obtaining a normal distribution before performing statistical analysis of the relevant phenotypes. All statistical analyses were performed using Statistica version 7.1 (StatSoft Inc. Tulsa, USA) and/or BioMedical Data Program (BMDP) version 1.12 (Los Angeles, USA).

## Results

We have genotyped SNPs rs1799969 R241G and rs5498 E469K in the ICAM-1 gene with the GoKinD material. For genotyping quality control, DNA samples of the patients with or without DN were distributed randomly across plates, and negative controls (water blanks) were included in each plate. Successful genotype calls in all samples were ≥95%, and the plates were randomly genotyped twice for duplication. The accuracy was calculated to be 98%. Genotype distribution in both was in HWE (P > 0.05).

We first examined the allelic association of SNPs rs1799969 R241G and rs5498 E469K in the ICAM-1 gene with DN in the GoKinD population. The frequencies of minor alleles in these two polymorphisms were similar between the groups of T1D patients with and without DN (Table 2). In SNP rs5498 E469K, the G allele frequency in the patients without DN was 46.0%, which was not significantly different from the frequency in the patients with DN (44.8%, P = 0.554). In SNP rs1799969 R241G, the A allele frequencies were 11.5% in T1D patients without DN and 10.3% in the patients with DN (P = 0.325). We further analyzed the heterozygosity and allele positivity of these two SNPs, and found that rs5498 E469K had high heterozygous index, and the difference of heterozygous frequencies between T1D patients with and without DN was borderline significant (P = 0.052). We found that the frequency of major allele A in female T1D with DN was similar to the frequency in female patients without DN (57.2% vs 54.2%, P = 0.265). The association of heterozygosity and allele G positivity between T1D patients with and without DN in females was found to be significant (P = 0.010, OR = 0.633, CI 95% 0.447–0.895 and P = 0.026, OR = 0.692, CI 95% 0.500–0.958, respectively), but not in males (P = 0.766 and 0.853). In the analyses, statistical power was calculated with number of patients, type 1 error probability for a two-sided test, relative risk of the control treatment relative to the experimental treatment and ratio of control to experiment patients, and the power reached to 0.9. In SNP rs1799969 R241G, there was no high heterozygous index in the genotype distribution. Analyses of allelic frequencies and genotype distributions in this polymorphism between the patients with and without DN in females, males or both sexes were performed and no significant association was found.

**Table 2:** Association of SNPs rs1799969 G241R and rs5498 E469K in the ICAM-1 gene between T1D with DN and T1D without DN

SNP ID	Position*	SNP type	Groups	Genotypes (%)			MAF	Heterozygous	Allele positivity
rs1799969	Exon 4	R241G	T1D without DN	AA	AG	GG			
	1657594	R = A/G	All	10 (1.61)	123 (19.84)	487 (78.55)	0.115		
			Male	3 (1.20)	52 (20.71)	196 (78.09)	0.116		
			Female	7 (1.90)	71 (19.24)	291 (78.86)	0.115		
			T1D with DN						
			All	3 (0.46)	130 (19.73)	526 (79.81)	0.103	*P *= 0.877	*P *= 0.576
			Male	1 (0.29)	77 (22.12)	270 (77.59)	0.114	*P *= 0.721	*P *= 0.884
			Female	2 (0.64)	53 (17.04)	256 (82.32)	0.092	*P *= 0.413	*P *= 0.258
rs5498	Exon 6	E469K	T1D without DN	GG	GA	AA			
	1658485	R = A/G	All	121 (19.58)	326 (52.75)	171 (27.67)	0.460		
			Male	50 (20.08))	130 (52.21)	69 (27.71)	0.462		
			Female	71 (19.24)	196 (53.12)	102 (27.64)	0.458		
			T1D with DN						
			All	141 (21.30)	311 (46.98)	210 (31.72)	0.448	*P *= 0.052	*P *= 0.113
			Male	75 (21.43)	176 (50.28)	99 (28.29)	0.466	*P *= 0.766	*P *= 0.853
			Female	66 (21.15)	135 (43.27)	111 (35.58)	0.428	*P *= 0.010¤	*P *= 0.026#

*Contig accession number is NT_011295. T1D = type 1 diabetic patients, and DN = diabetic nephropathy. MAF = minor allele frequency.

¤Odd ratio = 0.633 (CI 95% 0.447–0.895); #For the G allele Odd ratio = 0.692 (CI 95% 0.500–0.958).

We attempted to detect the association between phenotypes and genotypes of SNP rs5498 E469K in the female T1D patients without DN or with DN. We found no significant difference in clinical features among female T1D patients with DN carrying three different genotypes, although the patients with DN and genotype A/G had slightly increased diastolic blood pressure in comparison with the patients with homozygous genotypes A/A and G/G (74 ± 11 vs 71 ± 11, 71 ± 11 mmHg, P = 0.063, Table 3). Among the female T1D patients without DN carrying genotypes A/A, A/G and G/G, however, there were significant differences in cystatin levels (0.79 ± 0.17, 0.81 ± 0.14 and 0.75 ± 0.12 mg/L, P = 0.021).

**Table 3:** Clinical features of female T1D patients with or without DN according to genotypes of SNP rs5498 E469K

	T1D patients without DN			*P*-value	T1D patients with DN			*P*-value
				
Genotypes	AA	AG	GG		AA	AG	GG	
Age (yrs)	39 ± 8	39 ± 9	40 ± 9	0.631	43 ± 7	42 ± 8	43 ± 7	0.411
Duration (yrs)	26 ± 7	26 ± 8	27 ± 8	0.597	30 ± 9	31 ± 8	33 ± 8	0.211
HbA1c (%)	7.72 ± 1.27	7.50 ± 1.12	7.33 ± 0.89	0.070	7.54 ± 2.14	7.26 ± 1.99	7.20 ± 2.15	0.475
BMI (kg/m^2^)	25.6 ± 4.9	25.8 ± 4.6	25.1 ± 4.2	0.527	26.1 ± 6.3	25.2 ± 6.1	24.9 ± 4.9	0.308
Cystatin (mg/L)	0.79 ± 0.17	0.81 ± 0.14	0.75 ± 0.12	0.021	2.20 ± 1.86	2.38 ± 1.92	2.18 ± 1.64	0.690
Creatinine (mg/dL)	0.82 ± 0.14	0.85 ± 0.14	0.81 ± 0.13	0.111	2.02 ± 2.29	2.11 ± 1.90	2.01 ± 1.96	0.932
Cholesterol (mg/dL)	185.9 ± 29.3	190.2 ± 31.8	189.1 ± 33.0	0.569	185.2 ± 45.8	193.2 ± 45.0	189.8 ± 47.7	0.424
HDL (mg/dL)	63.8 ± 15.1	65.0 ± 15.6	65.0 ± 15.8	0.809	58.5 ± 17.4	59.4 ± 20.0	58.4 ± 17.3	0.904
SBP (mmHg)	116 ± 13	116 ± 11	117 ± 13	0.755	130 ± 21	132 ± 20	128 ± 20	0.472
DBP (mmHg)	71 ± 8	69 ± 7	70 ± 7	0.221	71 ± 11	74 ± 11	71 ± 11	0.063

Data are means ± SD. T1D = type 1 diabetic patients and DN = diabetic nephropathy.

## Discussion

In the present study, we have genotyped two non-synonymous SNPs rs1799969 R241G and rs5498 E469K in the ICAM-1 gene in a relatively large cohort from the GoKinD study. This cohort consists of the cases of T1D patients with severity of DN and the controls of T1D patients without DN. Data indicated that SNP rs5498 E469K presented a high heterozygous index in the genotype distribution of this GoKinD population. The observation is consistent with the previous and our recent studies in Danish, Finnish, Japanese and Swedish populations (See the summary, 19). Interestingly, we found that the major allele A in SNP rs5498 E469K was increased from T1D patients without DN to the patients with DN in females of the GoKinD population. Further analyses of heterozygosity and allele positivity indicated that the minor allele G of this SNP was associated with decreased risk susceptibility to the development of DN in female T1D patients. In our previous study using Swedish material [19], the gradually increasing of the major allele A of this polymorphism from non-diabetic subjects, to T1D patients without DN and to the patients with DN was observed. The association between this polymorphism and DN was not detected most likely due to limited size of samples.

We have attempted to detect the association of phenotypes according to the genotypes of this SNP in T1D patients with DN. We found no significant difference in clinical features among female T1D patients with DN carrying three different genotypes. Among the female patients without DN, however, the carriers with heterozygosity had significantly increased serum cystatin levels than the patients with heterozygosity. The data indicate that the heterozygosity of SNP rs5498 E469K may play a role in the development of DN. The previous studies demonstrate that the allele A of SNP rs5498 E469K is increased in T1D patients compared to non-diabetic subjects [14,15,19]. In the present study, we found that the allele G of this SNP is associated with the decreased risk susceptibility in female T1D patients with DN. Question concerning the gender specificity of genetic influence of the ICAM-1 genetic variation is then taken into our consideration. Beyond this study, we have recently studied several candidate genes, including NPY and AdipoQ in T1D and DN, and observed that the genetic polymorphisms in the NPY and AdipoQ genes are associated with T1D patients with DN in females but not in males [23,24]. However, we can't simply make a conclusion because our knowledge needs to be improved based upon accumulation of evidence from genetic studies and clinical observation in relation to gender specificity of DN.

rs1799969 R241G(G/A) is another non-synonymous polymorphism in the ICAM-1 gene and its genotype distribution has no high heterozygous index in the GoKinD population. Previous reports demonstrate that the allele G of this SNP is transmitted in T1D families among Finnish, British, Romanian, European and American Caucasians [18]. But, this polymorphism is not associated with DN among T1D patients in either Swedish [19] or GoKinD populations. A recent study has indicated that SNP rs5498 E469K but not rs1799969 R241G in the ICAM-1 gene is associated with the development of differentiation syndrome in actue promyelocytic leukemia [25]. Taking together with our study and the recent report, we suggest that SNP rs1799969 R241G may not be the major defect in the development of DN.

## Conclusion

In conclusion, we have evaluated the association between the ICAM-1 genetic polymorphisms and DN with the material from the GoKinD study and provided evidence that SNP E469K but not R249G is associated with DN in T1D. The allele G of SNP E469K in the ICAM-1 gene may confer the decreased risk susceptibility to the development of DN in female T1D patients.

## Abbreviations

DN: diabetic nephropathy; GoKinD: the Genetics of Kidneys in Diabetes study; ICAM-1: intercellular adhesion molecule-1; SNP: single nucleotide polymorphism; T1D: type 1 diabetes.

## Competing interests

The authors declare that they have no competing interests.

## Authors' contributions

JM carried out the molecular genetic studies and data analysis. DZ assisted with experiments and data analysis. KB, SE and HFG conceived of the study and also evaluated the data. HFG drafted the manuscript. All authors participated in the study design and coordination. All authors read and approved the final version of manuscript.

## Pre-publication history

The pre-publication history for this paper can be accessed here:


